# A Theoretical Review on EFL/ESL Teachers' Professional Development: Approaches, Applications, and Impacts

**DOI:** 10.3389/fpsyg.2022.912365

**Published:** 2022-05-27

**Authors:** Xiaodong Li

**Affiliations:** The Department of School of Foreign Studies, University of Science and Technology, Beijing, China

**Keywords:** professionalism, teacher professional development, educational systems, EFL/ESL teacher, L2 education

## Abstract

Teachers as the most important elements of education constantly need professional development (PD) courses in order to improve their pedagogy and practice. Given this, many educational systems worldwide have paid special attention to designing courses by which the quality of teaching and learning raises considerably. This surge of interest has ended in different studies on PD programs in L2 education. However, the pertinent literature lacks a comprehensive review of the models, applications, and impacts of EFL/ESL teachers' PD and various aspects influenced by this construct. To fill this gap and add fresh insights into this strand of research, the present study aimed to review the definitions, characteristics, models, goals, and uses of teacher professional development (TPD) in L2 education. Moreover, several empirical studies were touched on to support the claims of TPD impact on teachers. Finally, the study presented different implications for L2 teachers, teacher trainers, researchers, and policy-makers who can realize the significance and impact of effective TPD courses on the whole process of teaching and learning.

## Introduction

With the globalization and internationalization of education, English speaking users have been growing in number across the globe (Jenkins, 2000). This led to the generation of unique linguistic features (Englishes) and sociolinguistic realities that posed diverse challenges for English language teachers who had to prepare learners for performing well in the realities/complications ready outside the classroom walls (Akiba, 2013; Galloway and Rose, 2017; Matsuda, 2018). To respond to these calls, educational researchers, practitioners, principals, and policy-makers have been engaged in sententious debates on the criticality of “teacher quality” in improving students' academic performance and school success (Desimone, 2011; Macia and García, 2016; Sancar et al., 2021). To increase the quality of teaching, hence, different educational systems around the world devoted large amounts of time and budget to enhancing teachers' pedagogical effectiveness and quality by involving them in professional development (PD) programs (Guskey and Yoon, 2009; DeMonte, 2013; McChesney and Aldridge, 2018; Sancar et al., 2021). Additionally, the quick changes in language education mandated high pedagogical standards on the part of the teachers which, in turn, elevated expectations for teacher skills and professionalism (Bubb, 2004; Soebari and Aldridge, 2016; McChesney and Aldridge, 2018). Likewise, teachers' self-expectations intensified with the emergence of innovative educational trends (Collinson et al., 2009).

Obviously, the constant need for teachers to improve their instructional expertise highlighted a shift in PD programs which have long been transmission-focused interventions prescribed by school administrators to bridge the gaps in teachers' knowledge and skills by some activities (Broad and Evans, 2006; Desimone, 2009; McChesney and Aldridge, 2018). This deficit paradigm for teacher professional development (TPD) is now replaced by teacher-led PD that stresses the agency and active role of teachers in their own development (Lieberman and Miller, 2014). PD is no longer something done to teachers, but with teachers to cause pedagogical growth and academic success (Timperley et al., 2007; Avalos, 2011; Youngs and Lane, 2014). After the crystallization of the concept of PD, its features, and activities, many courses and programs worldwide were proposed to improve novice and experienced teachers' pedagogy and skills (Evans, 2014; King, 2014; Barrera-Pedemonte, 2016; McChesney and Aldridge, 2018). In a similar manner, an abundance of research has been done on effective TPD programs making considerable advances in the theoretical and practical underpinnings of this domain (Timperley et al., 2007; Muijs and Lindsay, 2008; Barrera-Pedemonte, 2016; Opfer, 2016; McChesney and Aldridge, 2018). More particularly, the results of investigations pinpointed that PD is a key element of teacher education which is helpful for different stakeholders in case its requirements are met (Borg, 2018; Haug and Mork, 2021). Moreover, scholars figured out different perceptions and beliefs concerning TPD and its impacts on various academic zones (e.g., Bett and Makewa, 2018; Griffin et al., 2018; Gutierrez-Cobo et al., 2019; Liu and Phelps, 2020; Christoforidou and Kyriakides, 2021).

Nonetheless, to many educators and scholars, TPD is still a murky concept due to the multi-layered nature of the construct (Sancar et al., 2021). PD programs are context-specific and an effective program may work poorly in other settings (Barrera-Pedemonte, 2016). Moreover, teachers and administrators may hold different views concerning the elements of an effective TPD, its features, practices, scope, and scientific propositions (Muijs and Lindsay, 2008; Opfer, 2016; McChesney and Aldridge, 2018; Komba and Mwakabenga, 2019). Correspondingly, many existing studies on TPD do not have a clear sense of the concept, its dimensions, frameworks, characteristics, manifestations, and holistic essence (Sales et al., 2011; Evans, 2014; Bett and Makewa, 2018). This is ironic in that educators must first understand TPD and the processes through which teachers grow professionally as well as the conditions that maintain and improve that growth before designing PD courses for teachers (Clarke and Hollingsworth, 2002; Korthagen, 2017; Komba and Mwakabenga, 2019). Motivated by these shortcomings, the present study aimed to review the conceptualizations, models, features, approaches, benefits, and impacts of TPD programs in the context of second/foreign language education and contribute to the theoretical and empirical bases of the field.

## Background

### Professionalism and Professional Development

The concepts of professionalism and PD as key elements of success and quality in various jobs have attracted a great deal of attention over the past few decades (Borg, 2018; Liu and Phelps, 2020). The term PD, in education, refers to various actions, activities, and processes that are designed for teachers in order to promote their teaching skills, knowledge, and attitudes as well as causing students' learning (Guskey, 2000; Avalos, 2011). Such programs are insightful in that in academic settings, competent students are made only if the teachers, as pillars, have received sufficient training for teaching efficiently (Yan, 2021). PD includes different educational experiences related to one's work (Mizell, 2010; Christoforidou and Kyriakides, 2021). It can be formal like conferences, seminars, workshops, and informal like discussions among work colleagues, reading and research, observations of others' work, or learning from a peer (Arthur, 2016; Petty et al., 2016). It is an on-going and context-sensitive attempt that can help teachers not only to improve their pedagogical knowledge but also provides a forum through which they can share their experiences with others (Creese et al., 2013; Sancar et al., 2021).

Similarly, Richards and Farrell (2005) argued that PD intends to help teachers understand teaching and their role as a teacher. For Craft (2000), TPD is a sort of modification by which teachers need a blend of engagement, support, pressure, and success. The final modification attained during or after the PD contributes to the organization, curriculum, and learners. Furthermore, PD has been dismissed as a bottom-down approach in which something is done *with* the teachers rather than *to* them to improve their knowledge and skills in different areas (Timperley et al., 2007). In simple terms, TPD is any activity or consistent process intended to promote teachers' beliefs, attitudes, knowledge, and classroom practice as a key factor in the quality of students' learning (DeMonte, 2013).

### The Purposes of Teacher Professional Development

As a fundamental part of teacher education, TPD has long sought to obtain some goals in academic milieus. It has been like a tool to enhance teachers' professional abilities and attitudes, craft better schools, and eventually develop the learning process and student achievements (Carney et al., 2019; Haug and Mork, 2021). Likewise, Evans (2011) contended that the purpose of TPD programs is to change teachers' professional thinking, knowing, feeling, and doing. It has also been regarded as an opportunity to cause teachers' pedagogical change, positive professional growth, and support their personal and socio-emotional development (Guskey, 2002; Borg, 2018; Rodriguez et al., 2020). It is essential to teachers' ability to cope with educational innovation and manage various socioeconomic affairs internal and external to the school (Omar, 2014). Moreover, Bredeson (2002) perceived the purpose of TPD as to strengthen teachers' individual and collective practice. For others, TPD is provided to augment scientific and inquiry-based teaching (e.g., Capps et al., 2012) or to apply curriculum innovation (e.g., Visser et al., 2010). The fulfillment of the needs of the teachers and students is another goal of such programs (Middlewood et al., 2005). Some TPD courses are delivered to teachers of various experiences only to enhance their pedagogical awareness and practice (Setiawan and Kuswandono, 2020).

### Characteristics of an Effective TPD

Given the prominence of PD and TPD in L2 education, a large body of research has been conducted to unravel the key characteristics of an effective TPD (Loucks-Horsley et al., 2009; Visser et al., 2010; Omar, 2014; Borg, 2018; Setiawan and Kuswandono, 2020; Haug and Mork, 2021; Yan, 2021). Despite the multitude of research, there has been a consensus regarding what an operative TPD program is among researchers in this line of inquiry. As a case in point, Visser et al. (2010) maintained that an effective TPD program must be able to prepare teachers to implement curriculum innovation, offer them sufficient opportunities to develop science content, instructional strategies, and assessment methods, and facilitate collaboration among colleagues in the institution. They should encompass and cover teaching and learning challenges and difficulties and discuss the elements of a good practice.

Likewise, in a meta-analysis, Capps et al. (2012) considered TPD as effective in case it encourages scientific and inquiry-based teaching and generates authentic experiences for the teachers. They also added some other core features; being coherent with standards, lesson development, inquiry modeling, reflection, transference, and content and pedagogical knowledge. Effective PD provides sufficient time and resources; encourages collegial and collaborative exchange; provides procedures for assessing the PD experience; and is school-based (Wei et al., 2009). For Sims and Fletcher-Wood (2021), a good TPD is continuous, collective, practice-based, subject-specific, and draws on external expertise.

PD courses in which the teacher has agency and active roles in his/her development (Fitri et al., 2021) can bring about more sustainable academic outcomes for teachers and students. Additionally, good PD programs have the potential to increase teachers' classroom practices and their subject-specific knowledge (Appleton, 2008).

Moreover, as Park Rogers et al. (2010) argued, if the chief goal of a PD is to increase student learning, it must take into account the learning needs of students. In so doing, PD facilitators must train teachers on how to use student data to inform and improve their teaching practice. They need to show teachers how to design appropriate assessments, diagnose student needs, and constantly modify a standards-based curriculum to fulfill students' specific learning needs. Finally, as pinpointed by Price (2011), TPD can lead to practical changes in teachers and education for as long as they are informed by action research which is an integral component of an effective PD.

### Theoretical Models and Approaches of TPD

In their landmark study, Calderhead and Shorrock (1997) clustered the most widely used models of TPD into five categories each focusing on a specific aspect of learning how to teach. They included: (1) enculturation or socialization into the professional culture which considered learning as the induction into the school's prevailing values and practices, (2) the technology or knowledge and skills model which stresses on teachers' obtained knowledge and skills that lead to successful education, (3) subject knowledge and pedagogical content knowledge that help teachers communicate the subject matter that they are teaching, (4) moral endeavor model which regards teaching as a job that considers students and their interest, prepare them to take social roles, and is a co-constructed and delivered process, and (5) models that highlight the close relationship between teachers' personal and professional lives. This category increases teachers' contextual awareness and enables them to detect and preserve the moral and ethical issues embedded in their practice.

There are other models for TPD in the pertinent literature, as well. For instance, Clarke and Hollingsworth (2002) proposed an interconnected and non-linear model for PD in which PD was claimed to consist of four domains; personal, external, practical, and consequential. They maintained that changes in one domain may be reflected in others and such changes are mediated by reflection and enaction. Furthermore, Desimone (2009) proposed five features of PD including content focus, active learning, coherence, duration, and collective participation. He also argued that there is an interactive, non-recursive relationship among these five critical features. In his model, Desimon claimed that a change in teachers' beliefs will happen after experiencing a PD program that persuades the teacher to acquire a new skill and knowledge that eventually will heighten students' learning. Evans (2014) introduced a model that focused on the concept or the process of PD. He argued that the TPD process includes behavioral, attitudinal and intellectual developments on the part of the teachers.

Practically, TPD programs can take different approaches. They can be *supportive* (identify the burden, obligation, and interest of teachers and schools), *job-embedded* (deal with teachers' daily responsibilities), *instructionally focused* (focus on students' learning outcomes), *collaborative* (involve teachers in both active and interactive learning), and *ongoing* which is a mixture of contact hours, duration, and coherence. Based on this approach, the more the teachers engage in PD, the more likely their pedagogy is to advance (Setiawan and Kuswandono, 2020). The problem with these models is that they are not specialized to L2 education, present TPD in a linear fashion of stages/levels, and offer PD activities applicable in a particular context/discipline. Hence, it is assumed that second/foreign language education needs discipline-specific models so that EFL/ESL teachers can grow professionally using scientific and well-designed stages and activities.

### The Applications and Merits of TPD

The main benefit of offering or participating in TPD programs is functioning as mechanism for the improvement of teachers' theoretical and practical knowledge and their teaching expertise (Borg, 2018; Setiawan and Kuswandono, 2020). With the spread of English and L2 education, the need for enhancing the quality of education raised to meet various academic standards and this demanded a deep focus on the delivery of effective PD courses for instructors by teacher trainers, scholars, educational institutions, and other stakeholders (Soebari and Aldridge, 2016; McChesney and Aldridge, 2018).

Likewise, Bolam and Weindling (2006) pinpointed that well-designed TPD programs can cause fundamental changes in teachers' practice, school quality, and students' achievement. Furthermore, as research certifies, teachers' knowledge develops after taking part in TPD courses (Wilde, 2005; Dalgarno and Colgan, 2007; McNicholl and Noone, 2007; Miller and Glover, 2007; Borg, 2018; Setiawan and Kuswandono, 2020). Also, other studies demonstrated that teachers' pedagogical beliefs and attitudes can change and develop as a result of teacher training (Pedder, 2006; Avalos, 2011; DeMonte, 2013). Additionally, TPD courses have been found to modify and promote teachers' teaching style, planning, and assessment (Boyle et al., 2004; Christoforidou and Kyriakides, 2021).

Moreover, as put by Cordingley et al. (2005), TPD courses are meritorious to educational institutions at any level, be it in the primary, middle school, high school or even the university level. Teachers constantly require regular instruction in their profession and related subjects if they are to develop their teaching attitudes, beliefs, and pedagogical practices in the classroom. Such programs contribute to teachers' development of teaching skills and content knowledge that, in turn, culminate in students' learning and school effectiveness (Carney et al., 2019). There may be many other applications in the use of TPD in various fields and academic domains that demand further investigations and involvement of insiders' and outsiders' perspectives.

### The Impacts of TPD on Second/Foreign Language Education

TPD can work as a catalyst of change in that many aspects of L2 education can be affected by this construct. As a case in point, many studies in the literature, as mentioned earlier, approved the impact of TPD programs on EFL teachers' pedagogical practices, content knowledge, perceptions, experiences, attitudes, classroom behaviors, and the like. Others referred to the role of such programs in developing teachers' sense of self-efficacy (Ross and Bruce, 2007), satisfaction (O'Sullivan, 2011), teaching quality (Bicaj and Treska, 2014), professional identity (Garner et al., 2016), positional identity (Moore, 2008) and so forth. As for students, TPD has been identified to increase students' academic gains/achievements, as well (Guo and Yang, 2012; Haug and Mork, 2021). There are many other changes in L2 teaching and learning landscapes under the influence of effective PD courses including materials development, assessment, teacher education, institutions, etc. Finally, PD and courses designed to improve teachers and teaching are claimed to affect the research-engaged levels of teachers and trainers (Borg, 2010; Dengerink et al., 2015).

### Concluding Remarks

In this review article, it was argued that in the context of second/foreign language teaching and learning PD has a crucial role. It can influence various aspects of education if it is delivered efficiently. This study went through the main features, models, approaches, uses, and effects of TPD in English language teaching to spark more light on the criticality of teacher quality in academia. As a result, the article can be insightful for EFL/ESL teachers in that they understand the role and power of TPD programs in their career, practice, and identity. They can attend many pre-service and in-service courses to constantly develop their professional skills and abilities in tune with the academic objectives of a given context. Teacher educators can also find this review beneficial in that they can get familiar with the core features of successful and effective TPD programs and apply such ideas in their future training courses. When they know the elements of TPD programs, they can offer techniques and strategies to novice teachers to grow in their job. Based on this review, there are many areas affected by TPD, hence teacher educators need to propose courses in which teachers are actively engaged in/with the program instead of being mere listeners to instructional suggestions and techniques.

Moreover, policy-makers and curriculum designers can peruse this study and realize the importance of TPD courses for all teachers regardless of their disciplines. Hence, they can revisit their plans and outlooks as per teacher education, especially in L2 education. In a similar manner, L2 researchers can find this review study beneficial and run further studies to add to the body of knowledge in this domain and bridge the existing gaps. As reviewed, many of the models in the literature follow a linear process or path applicable in a specific context. Hence, future scholars can develop models which are more activity and practice-oriented so that teachers from different parts of the world can use them and professionally develop. Likewise, a recent approach or model can be designed and tested by avid researchers to fulfill the needs of teachers and researchers in Applied Linguistics. Another gap is that TPD research has mostly focused on teaching and the effect of such programs on language assessment has been overlooked. Therefore, future research can be done on the impact of training courses on various assessment perceptions and practices of EFL teachers. Additionally, designing a reliable and valid tool to measure the effectiveness of TPD can also be an interesting area for research. All in all, it should be stated that research on TPD is rapidly growing and many insightful findings have been obtained in L2 education. However, there are still unexplored avenues and ignored angles demanding further scholarly attempts.

## Author Contributions

The author confirms being the sole contributor of this work and has approved it for publication.

## Funding

This study is sponsored by 2017 Project of Young Excellent and Talent Scholars of University of Science and Technology Beijing. It is part of the outcomes of the project Research on flipped classroom of college English education based on pan learning strategy (Project No. 06200041), which is sponsored by Central higher education fundamental research funds. It is also part of the outcomes of USTB Massive Online Open Course Project (Project No. KC2021ZXKF21). This study is also funded by The 14th Five Year Planned Teaching Material of USTB (Project No. JC2022YB037).

## Conflict of Interest

The author declares that the research was conducted in the absence of any commercial or financial relationships that could be construed as a potential conflict of interest.

## Publisher's Note

All claims expressed in this article are solely those of the authors and do not necessarily represent those of their affiliated organizations, or those of the publisher, the editors and the reviewers. Any product that may be evaluated in this article, or claim that may be made by its manufacturer, is not guaranteed or endorsed by the publisher.
